# The Posterior Part Influences the Anterior Part of the Mouse Cranial Base Development

**DOI:** 10.1002/jbm4.10589

**Published:** 2021-12-24

**Authors:** Honghao Zhang, Ke'ale W Louie, Anshul K Kulkarni, Karen Zapien‐Guerra, Jingwen Yang, Yuji Mishina

**Affiliations:** ^1^ Department of Biologic and Materials Sciences & Prosthodontics, School of Dentistry University of Michigan Ann Arbor MI USA

**Keywords:** HEDGEHOGS, CELL/TISSUE SIGNALING—PARACRINE PATHWAYS, CHONDROCYTE AND CARTILAGE BIOLOGY, GROWTH PLATE, CHONDROCYTE AND CARTILAGE BIOLOGY, DENTAL BIOLOGY, DEVELOPMENTAL MODELING, BONE MODELING AND REMODELING

## Abstract

The cranial base is a critical structure in the head, which is composed of endoskeletal and dermal skeletal. The braincase floor, part of the cranial base, is a midline structure of the head. Because it is a midline structure connecting the posterior skull with the facial region, braincase floor is critical for the orientation of the facial structure. Shortened braincase floor leads to mid‐facial hypoplasia and malocclusions. During embryonic development, elongation of the braincase floor occurs through endochondral ossification in the parachordal cartilage, hypophyseal cartilage, and trabecular cartilage, which leads to formation of basioccipital (BO), basisphenoid (BS), and presphenoid (PS) bones, respectively. Currently, little is known about whether maturation of parachordal cartilage, hypophyseal cartilage, and trabecular cartilage occurs in a simultaneous or sequential manner and if the formation of one impacts the others. Our previous studies demonstrated that loss of function of ciliary protein *Evc2* leads to premature fusion in the intersphenoid synchondrosis (ISS). In this study, we take advantage of *Evc2* mutant mice to delineate the mechanism governing synchondrosis formation. Our analysis supports a cascade mechanism on the spatiotemporal regulation of the braincase floor development that the hypertrophy of parachordal cartilage (posterior side) impacts the hypertrophy of hypophyseal cartilage (middle) and trabecular cartilage (anterior side) in a sequential manner. The cascade mechanism well explains the premature fusion of the ISS in *Evc2* mutant mice and is instructive to understand the specifically shortened anterior end of the braincase floor in various types of genetic syndromes. © 2021 The Authors. *JBMR Plus* published by Wiley Periodicals LLC on behalf of American Society for Bone and Mineral Research.

## Introduction

**Fig. 1:**
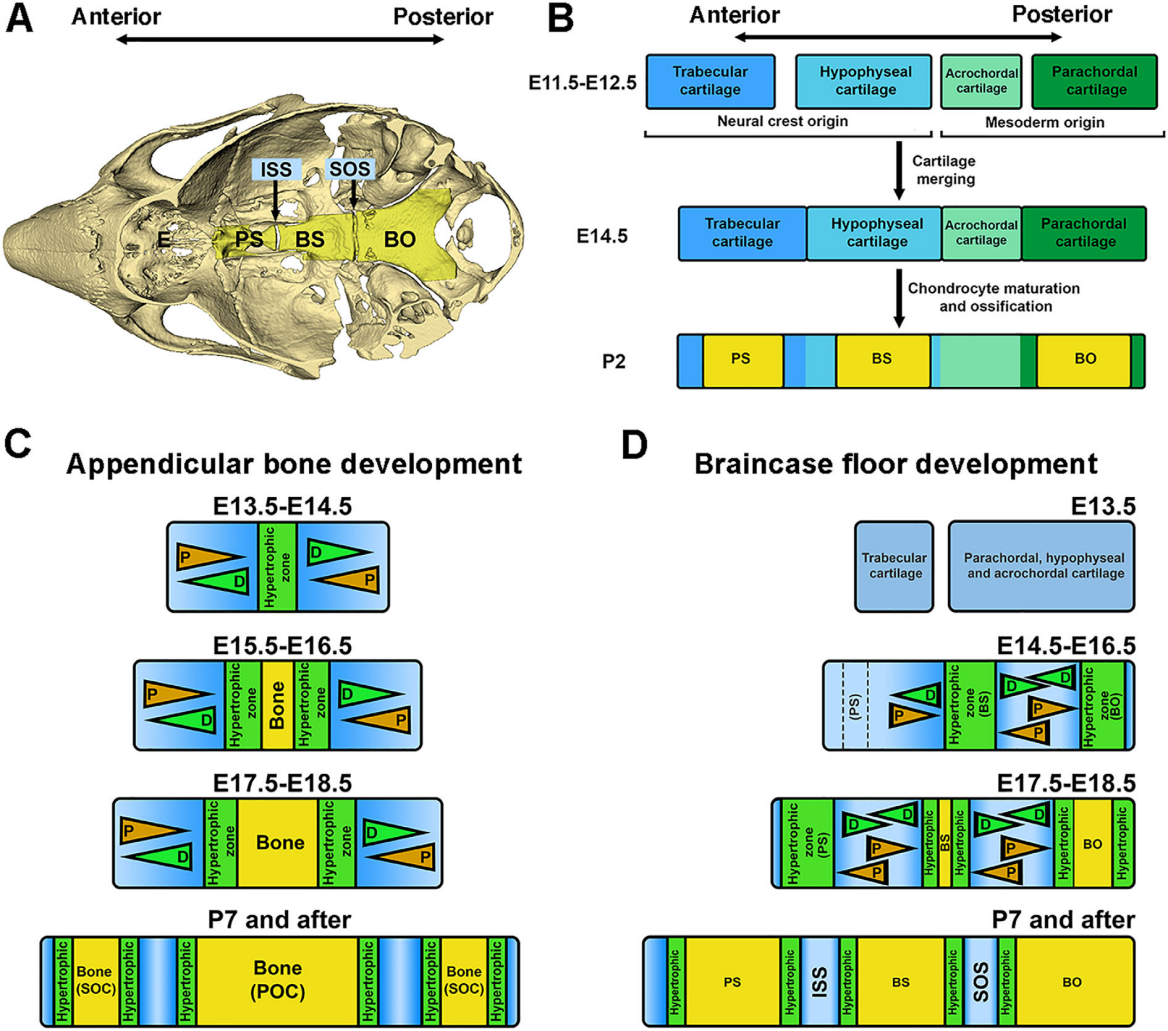
A cascade model of braincase floor elongation summarized from studies in this report. (*A*) A mouse skull model indicates the anatomic location of the braincase floor. ISS = the intersphenoid synchondrosis; SOS = the spheno‐occipital synchondrosis; PS = presphenoid bone; BS = basisphenoid bone; BO = basioccipital bone. (*B*) Braincase floor development described in previous studies is summarized. Continuous braincase floor is the result of fusion of four cartilages, the parachordal, acrochordal, hypophyseal, and trabecular cartilage from the posterior to the anterior. Then hypertrophic differentiation and subsequent mannerization within parachordal, hypophyseal, and trabecular cartilage leads to formation of BO, BS, and PS in the braincase floor. (*C*) A model describes appendicular bone elongation. Initial hypertrophy occurs at the middle of cartilage primordia. The growth plate flanking the hypertrophic zone leads to bidirectional elongation of the appendicular bone. (*D*) A hypothetical model of how the braincase floor elongates. Chondrocytes located at the most posterior end of the braincase floor cartilage primordia start to differentiate to form the hypertrophic zone due to an activity of the remnant notochord, which eventually give rise to the BO. Hypertrophic chondrocytes in this zone subsequently induce differentiation of chondrocytes at the middle of the braincase floor cartilage primordia to form the second hypertrophic zone, which is the future BS, and finally, hypertrophy of the chondrocyte zone for the future PS occurs at the anterior end of the braincase floor cartilage primordia. Three hypertrophic zones will be mineralized when embryogenesis progresses from posterior to anterior, and remaining cartilage will form two mirror‐imaged growth plates, the ISS between PS and BS and the SOS between BS and BO. These synchondroses support the bidirectional growth of each bone, which contributes to the elongation of the braincase floor. D = differentiation; P = proliferation.

The cranial base is a skeleton structure that separates brain from other facial structures. Anatomically located underneath the brain, the cranial base protects and supports the brain, pituitary, and sensory organs and connects to the trunk via the vertebral column.^(^ ^1^, ^2^, ^3^ ^)^ Historically, “cranial base” has been used to refer to the basioccipital (BO), the basisphenoid (BS), and the presphenoid (PS) bones and cartilage synchondrosis, the spheno‐occipital synchondrosis (SOS), and the intersphenoid synchondrosis (ISS), between BO and BS, and between BS and PS, respectively. As summarized and pointed out by recent reports, cranial base includes the above‐mentioned structures as well as other associated dermal skeletons.^(^ ^4^, ^5^ ^)^ To remain precise in the nomenclature, we will use “braincase floor” to refer the midline structure consisting of the BO, BS, PS, ISS, and SOS, which connects the posterior skull with facial region (Fig. 1*A*). Abnormally shortened braincase floor leads to mid‐facial hypoplasia and malocclusions, signs often observed in patients with syndromic disorders such as Down syndrome, Crouzon syndrome, and Pfeiffer syndrome.^(^ ^6^, ^7^, ^8^ ^)^ Elongations of the braincase floor and appendicular bone occur through similar mechanisms. However, knowledge from appendicular bones is not sufficient to cover all aspects of braincase floor development since elongation of the braincase floor supported by synchondrosis is bidirectional in nature. Understanding intricacies of the braincase floor development will provide fundamental insight and deepened appreciation regarding the pathophysiology underlying various abnormalities in craniofacial development.

The braincase floor development is through endochondral ossification, in which cartilage primordia forms first, followed by mineralization. During embryonic development, braincase floor resulted from fusion of four cartilages, the parachordal, acrochordal, hypophyseal, and trabecular cartilage from the posterior to the anterior (summarized in Fig. 1*B*).^(^ ^5^, ^9^, ^10^ ^)^ Although still in debate, it is commonly believed that the posterior part (parachordal and acrochordal cartilage) derived from the paraxial mesoderm and the anterior part (hypophyseal and trabecular cartilage) derived from cranial neural crest.^(^ ^10^, ^11^ ^)^ Later, all four cartilages fuse into a continuous structure. Hypertrophy within the parachordal cartilage, hypophyseal cartilage, and trabecular cartilage leads to formation of primordia of the BO, BS, and PS bones.^(^ ^5^, ^10^ ^)^ Elongation of the braincase floor is powered by two synchondroses, the ISS between the PS and BS, and the SOS between the BS and BO.^(^ ^12^, ^13^ ^)^

It is currently unknown whether hypertrophy of the parachordal, hypophyseal, and trabecular cartilages occurs in a simultaneous or sequential manner and if formation of each hypertrophic zone is dependent or independent on others. In contrast to appendicular bone development (summarized in Fig. 1*C*), existing evidence suggests that the braincase floor is structurally distinct from the growth plate in appendicular bones.^(^ ^8^, ^13^ ^)^ Formation of the hypertrophy of the parachordal cartilage during early embryonic stages is induced by *Shh* expressed in the notochord remnants, located next to the posterior end of the braincase floor.^(^ ^14^ ^)^ Radiologic studies have demonstrated that mineralization of the braincase floor extends from the posterior to the anterior end.^(^ ^15^ ^)^ Since mineralization occurs in hypertrophic zones, we hypothesized that the hypertrophy of parachordal, hypophyseal, and trabecular cartilages occurs in a sequential manner from posterior to anterior (Fig. 1*D*). Since chondrocyte hypertrophy leads to secretion of Indian Hedgehog (IHH) from prehypertrophic chondrocytes, which is critical for chondrocyte proliferation and differentiation, we hypothesized a cascade mechanism that hypertrophy of the parachordal cartilage induces the hypertrophy of the hypophyseal cartilage, which further induces hypertrophy of the trabecular cartilage during the braincase floor development (as summarized in Fig. 1*D*). Understanding the spatiotemporal orchestration of signaling cascades during synchondrosis patterning is critical for understanding proper braincase floor elongation and subsequent development of the midfacial region.

Ellis‐van Creveld syndrome (EVC) is an autosomal recessive chondrodysplasia.^(^ ^16^, ^17^, ^18^ ^)^ Our previous studies demonstrated that mid‐facial hypoplasia in *Evc2* (aka *Limbin*) mutant mice is not due to alterations in facial bones (maxilla and ethmoid) but rather due to a shortened braincase floor resulting from the premature fusion of the ISS observed as early as postnatal day 8 (P8).^(^ ^11^, ^19^ ^)^ Interestingly, *Evc2* loss of function leads to compromised, but not ablated, cellular response to Hedgehog ligand, thereby making it a valuable tool for delineating our cascade model during the braincase floor development. In this report, our comprehensive analysis of *Evc2* mutant braincase floors highlights how hypertrophy of the parachordal cartilage (posterior end) influences the maturation of the hypophyseal (middle) and the trabecular cartilage (anterior end) through progressive downregulation of Hedgehog signaling activities.

## Materials and Methods

### Animal models

Mice were maintained and used in compliance with the Institutional Animal Care and Use Committee (IACUC) of the University of Michigan in accordance with the National Institutes of Health Guidelines for Care and Use of Animals in research, and all experimental procedures were approved by the IACUC of the University of Michigan (protocol #PRO00009613). All mice were housed in a room with temperature between 18°C and 23°C with 40% to 60% humidity. *Evc2* mutant mice (*Evc2* ^*ex12/+*^) and *Evc2* floxed mice (*Evc2* ^*fl/+*^) were generated by our group as previously reported.^(^ ^20^ ^)^ Neural crest‐specific *Evc2* mutant mice were generated by crossing *Evc2* ^*fl/fl*^ mice with *Wnt1‐Cre* mice.^(^ ^21^ ^)^ All mice were maintained in a mixed background of C57BL6/J and 129S6 and were crossed and maintained in our semiclosed mouse colony for at least 5 years. For embryonic staging, the noon of identification of vaginal plug was E0.5. Consistent with findings in patients, studies from us and others provide molecular evidence that phenotypic abnormities due to *Evc2* loss of function passes through recessive inheritance.^(^ ^3^, ^20^ ^)^ For comparisons between genotypes, wild‐type mice and *Evc2* heterozygous mutant mice for the global knockout allele (*Evc2* ^*ex12/+*^) were used as controls to compare with global *Evc2* homozygous mutant mice (*Evc2* ^*ex12/ex12*^). For neural crest‐specific *Evc2* mutant mice (*Evc2* ^*fl/fl*^;*Wnt1‐Cre*), we compared with control littermates such as *Evc2* ^*fl/fl*^ or *Evc2* ^*+/fl*^;*Wnt1‐Cre* mice. Number of animals used for comparison per group are indicated as *n* in figure legends. Animals/embryos were included based on genotypes, and there are no animal/embryos excluded.

### Histology, immunohistochemistry, and in situ hybridizations

Braincase floors from postnatal or embryonic stages were dissected out, fixed in 4% paraformaldehyde (PFA), and decalcified in 14% EDTA. Subsequently, they were embedded in paraffin, sectioned parasagittally in 5 um thickness, and stained with hematoxylin and eosin (H&E) for histologic observations according to standard histology procedure. For histological quantifications, six represented sections per animal were evaluated. The average of six sections was used to represent the evaluated animal. The parameters measured were defined as following: presumptive BS: the length of hypertrophic zone for BS or BS with hypertrophic zone flanking BS; BS: the length of bony part of BS; presumptive BO: the length of hypertrophic zone for BO or BO with hypertrophic zone flanking BO; BO: the length of bony part of BO; total length of braincase floor: the length from the most anterior point to the most posterior point of the braincase floor; length of the anterior of the braincase floor: the length from the most anterior point to the most posterior point of the bony part of BS; BS + PS: the length from the anterior part of skeleton PS to the posterior part of skeleton BS. For 5‐ethynyl‐2′‐deoxyuridine (EdU) incorporation experiment, EdU was injected 3 hours before animal euthanization at 40 mg/kg. For immunohistochemistry, dissected braincase floors were fixed with 4% PFA and cryo‐protected by 30% sucrose in PBS before being embedded parasagittally for cryosection. Sections (10 μm thickness) were incubated overnight at 4°C with antibody against P‐Histone3 (PA5‐16183), OSX (ab22552, 1:500, Abcam, Cambridge, MA, USA), and RUNX2 (ab236639, 1:200, Abcam). Secondary antibody, anti‐rabbit IgG‐alexa fluor 488 (A‐32731) was from Life Technologies (Grand Island, NY, USA). Quantifications of number of cells with stained antibody were done with an average of four sections close to the midline to represent one biological sample. The average of four different biological samples was then taken for the comparison of differences between controls and mutants. Quantifications of staining intensity were done through ImageJ. In brief, for each sample, we assessed the fluorescence intensity in 20 cells in sections close to the midline. The average fluorescence intensity of 20 cells was used to represent one biological sample. For paired comparisons between controls and mutants, group comparison of four controls and four mutants were carried out. The anterior end of the ISS (ISS‐A), posterior end of the ISS (ISS‐P), the anterior end of the SOS (SOS‐A), and the posterior end of the SOS (SOS‐P) were examined. RNA in situ hybridization was carried out as previously described^(^ ^22^ ^)^ using digoxygenin‐labeled *Col10a1, Ihh, and Ptch1* probes.

### RNA isolation and quantitative real‐time PCR

Braincase floor synchondroses (ie, ISS‐A, ISS‐P, SOS‐A, and SOS‐P) were dissected out and split in the middle in anterior–posterior axis into two for the corresponding anterior and posterior portion before immediate homogenization in TRIzol (Life Technologies), according to manufacturer's instructions. For reverse transcription, 1 μg of total RNA was reverse‐transcribed using SuperScript Reverse Transcriptase (Life Technologies). Quantitative real‐time PCR (qRT‐PCR) was performed using Applied Biosystems (Carlsbad, CA, USA) ViiA7, with the following taqman probes: Mm00494645_m1 for *Gli1*, Mm99999915_g1 for *Gapdh*, Mm00439612_m1 for *Ihh*, Mm00436026_m1 for *Ptch1*, Mm00436057_m1 for *Pthrp*. For all analysis, RNA samples isolated from four controls and four mutants were used to compare.

### Primary chondrocyte isolation

Synchondroses from individual embryos were dissected from E17.5 embryos with all non‐cartilage tissues removed and digested with collagenase A (Roche, Indianapolis, IN, USA). Chondrocytes released were subsequently cultured in DMEM (Life Technologies) with 10% FBS (Atlanta Biologicals, Flowery Branch, GA, USA), and chondrocyte identity was verified according to previous report.^(^ ^23^ ^)^ The experiment was carried out using cells within five passages. Cells isolated from one embryo were designated as one line. Altogether there are three control lines, and three mutant lines were established for analysis presented. For induction of Hedgehog signaling, cultured primary chondrocytes were starved in 0.5% serum for 36 hours before treatment with 100 nmol of SAG (Chemicon, Billerica, MA, USA) for 24 hours before isolation of total RNA for qRT‐PCR. Hedgehog signaling induction was calculated by dividing post‐induction mRNA levels of Gli1 by pre‐induction levels.

### Statistical analysis

Paired *t* test was used for all studies presented and *t* test was performed in SPSS 27.0 (IBM Corp., Armonk, NY, USA). Error bars in the graph are standard deviation.

Additional data are available in the Appendix.

## Results

**Fig. 2:**
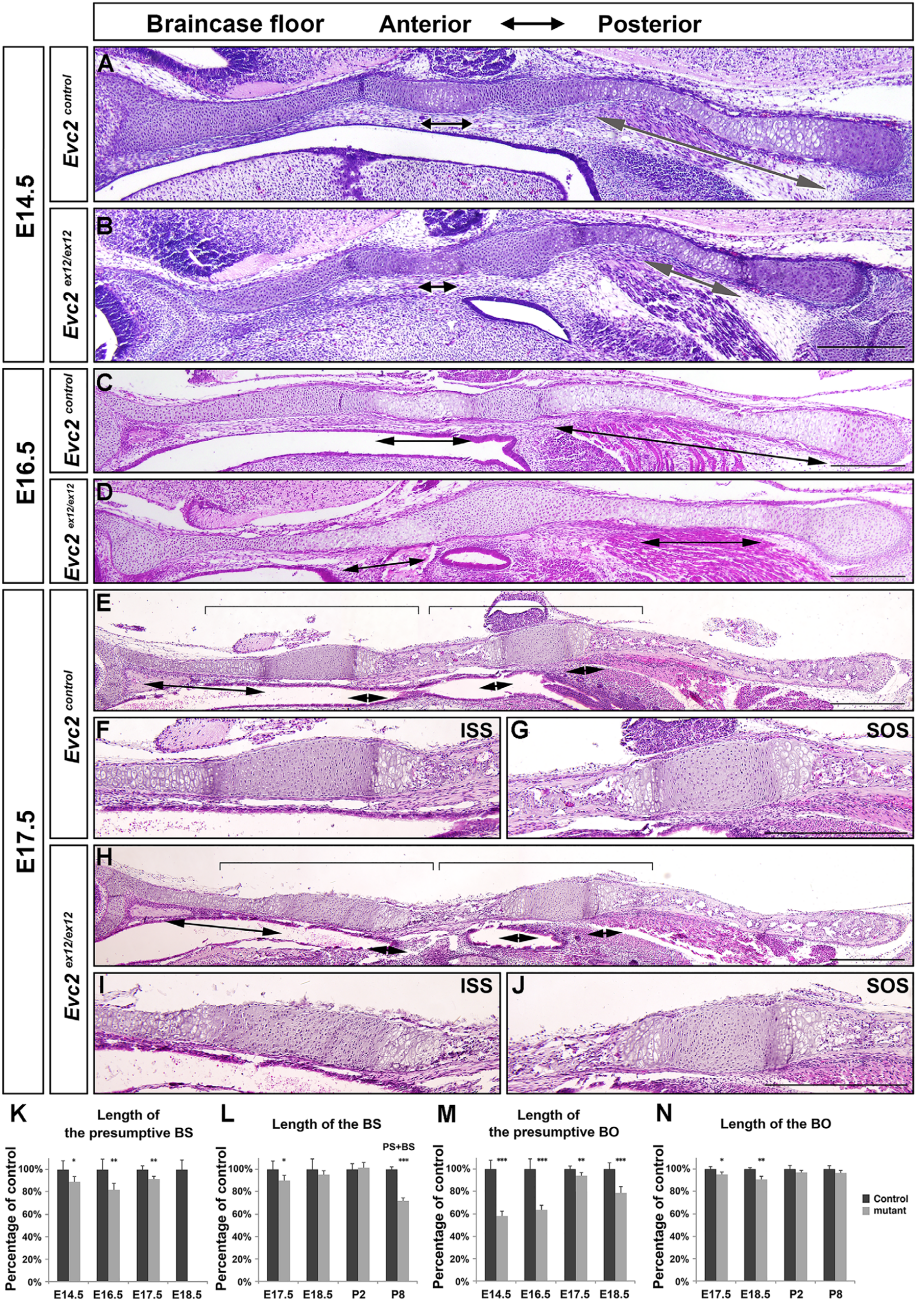
*Evc2* loss of function leads to braincase floor with abnormal structure before E18.5. Histological sections of control and *Evc2* mutant braincase floors at E14.5 (*A*, *B*), E16.5 (*C*, *D*), and E17.5 (*E*, *H*) were stained with H&E and shown. The enlarged ISS and SOS for E17.5 controls (*F*, *G*) and mutants (*I*, *J*) are shown. Brackets indicate enlarged region and double arrows indicate hypertrophic zones. Scale bar = 200 μm. Based on the histological assessment in control and *Evc2* mutant braincase floor, the length of the presumptive BS (*K*), length of the BS (*L*), length of the presumptive BO (*M*), and length of the BO (*N*) are quantified and shown as the percentage of controls. (*n* = 6, *p < 0.05, **p < 0.01, error bars denote standard deviations.)

Our investigation on the mechanisms governing the braincase floor elongation started from analysis of the braincase floor in *Evc2* mutants and controls at embryonic stages. *Evc2* global mutant allele (ex12) has a *LacZ* cassette inserted into the exon 12 of the endogenous *Evc2*. Through beta‐gal staining, we found that *Evc2* is expressed in nearly all cells in the mouse braincase floor^(^ ^20^ ^)^ (Fig. S1*A–F*). E13.0 is the earliest stage when cartilage is observed in the braincase floor during embryonic development.^(^ ^10^, ^24^ ^)^ At E13.5, we noticed a gap between the trabecular cartilage and the hypophyseal cartilage, suggesting that there is no continuous cartilage at the braincase floor (Fig. S2*A*). Levels of *Evc2* mRNA were comparable between the trabecular cartilage and rest of the three cartilages at E14.5, which were also comparable in ISS and SOS at E17.5 (Fig. S2*B*). At this stage, there was no morphological difference in the braincase floor of *Evc2* mutants (Fig. S2*A*). At E14.5 and E16.5, we observed hypertrophic zones for the parachordal (future BO) and the hypophyseal cartilage (future BS), respectively, in control braincase floors, but no hypertrophic zone for the trabecular cartilage (future PS) (Fig. 2*A–D*, double arrows). At these two stages, *Evc2* mutant braincase floors showed decreased lengths in hypertrophic zones in the parachordal (BO) and the hypophyseal (BS) cartilage (Fig. 2*K–M*), suggesting either decreased proliferation, accelerated maturation, or both in chondrocytes due to loss of *Evc2*. At E17.5, in controls, the hypertrophic zone in the trabecular cartilage developed in addition to the BO and the BS (Fig. 2*E–J*). We observed decreased lengths of the BO, BS, and the hypertrophic zone for the trabecular cartilage in *Evc2* mutant braincase floors (Fig. 2*K–N*). Overall, histological assessment demonstrated that hypertrophies of the parachordal cartilage (future BO) and the hypophyseal cartilage (future BS) initiates before E14.5, and hypertrophy of the trabecular cartilage (future PS) initiates before E17.5 in both control and mutant embryos.

**Fig. 3:**
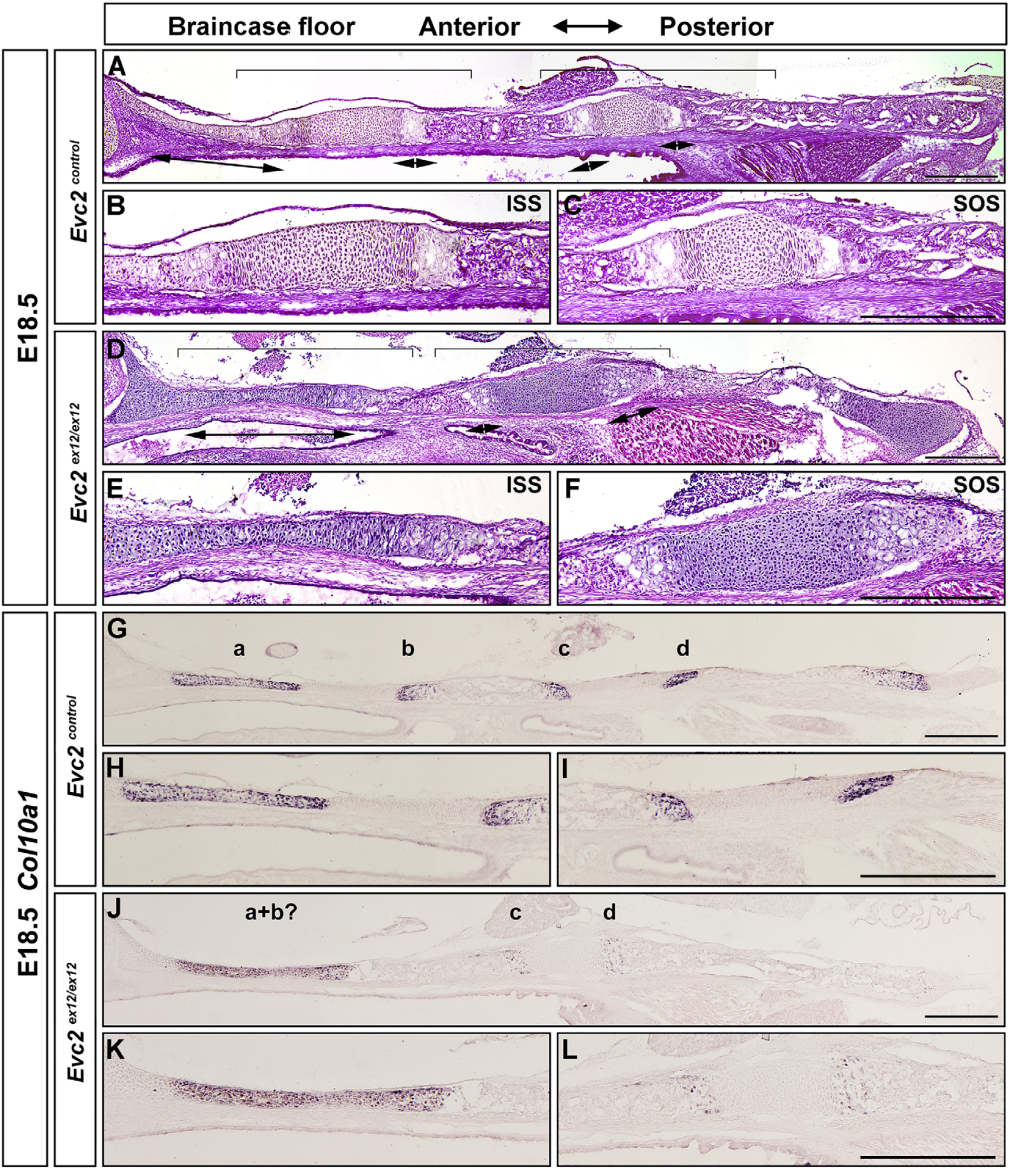
*Evc2* loss of function leads to shortened braincase floors and premature fusion of ISS at E18.5. Control and Evc2 mutant braincase floors at E18.5 (*A*, *D*) are shown. The enlarged ISS and SOS for controls (*B*, *C*) and mutants (*E*, *F*) are shown. Expressions of *Col10a1* in controls (*G–I*) and *Evc2* mutant (*J*–*L*) skull at E18.5 were examined by in situ hybridization. Hypertrophic zones for PS (*a*), ISS‐P (*b*), SOS‐A (*c*), and SOS‐P (*d*) are shown. The enlarged ISS and SOS for controls (*H*, *I*) and mutant (*K*, *L*) are shown. Pictures shown are representative of comparisons of 6 controls with mutants. Scale bar = 200 μm.

**Fig. 4:**
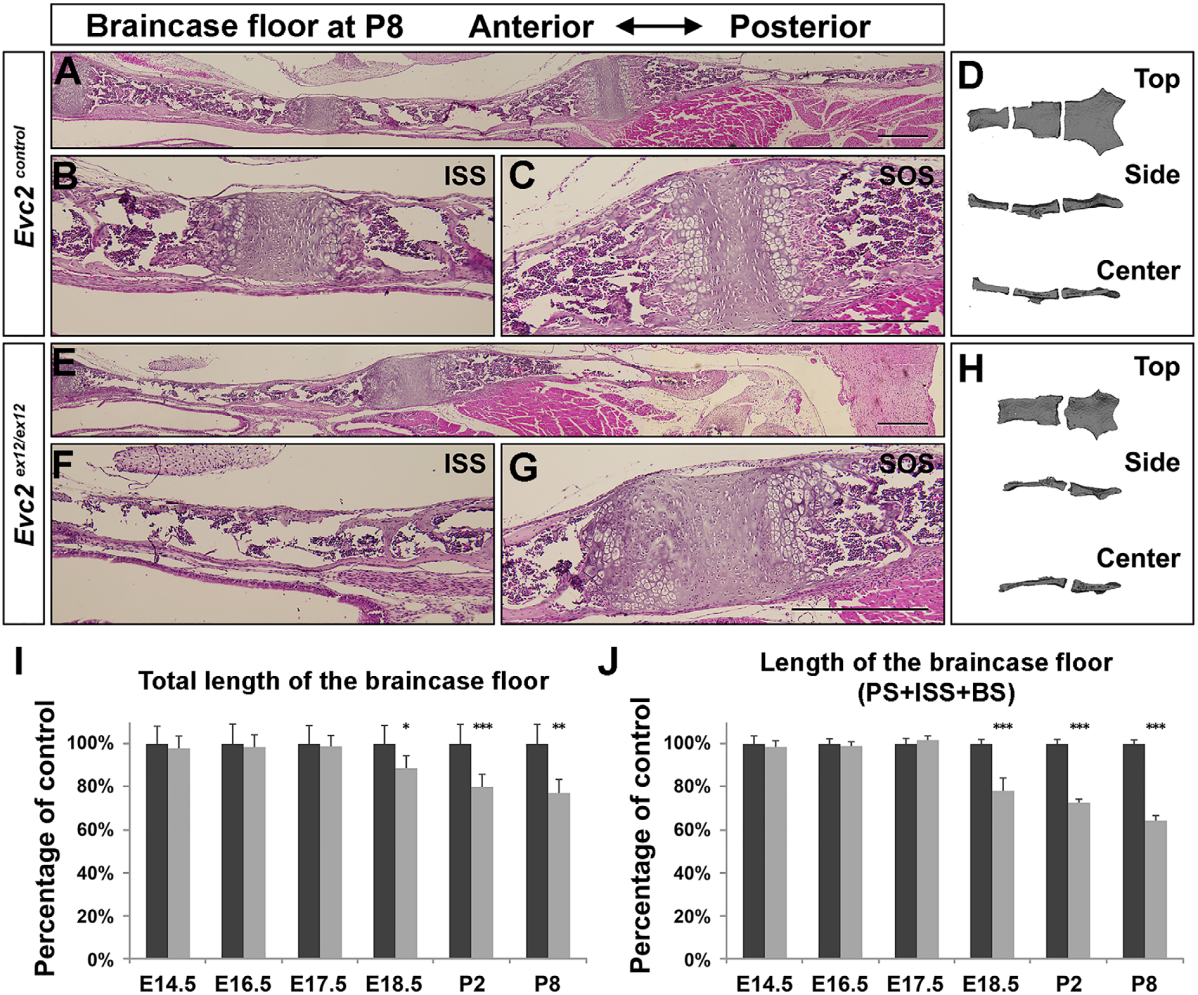
*Evc2* loss of function leads to fused ISS at P8. Histologic assessment of control (*A*) and *Evc2* mutant (*E*) skull bases at P8 are shown. The ISS of control (*B*), a presumptive ISS of mutant (*F*), and the SOS of control (*C*) and mutant (*G*) are shown. Three‐dimensional reconstructed models of control (*D*) and mutant (*H*) skull reconstructed from micro‐CT scan are shown. The length of the braincase floor (*I*) and length of the anterior braincase floor (*J*) are shown as the percentage of controls. (*n* = 6, *p < 0.05, **p < 0.01, error bars denote standard deviations.)

At E18.5, histological assessment demonstrated similar pattern of each zone in controls. In contrast, all cells in the ISS of *Evc2* mutants became hypertrophic chondrocytes (Fig. 3*A–F*). Hypertrophy of the entire ISS in *Evc2* mutants is consistent with the detection of *Col10a1* expression in the entire ISS in *Evc2* mutant braincase floors (Fig. 3*G–L*). At postnatal day 8 (P8), histological assessment indicated the absence of cartilage structure between PS and BS in *Evc2* mutant braincase floor (Fig. 4*A–C*, *E–G*). Consistently, μCT analysis confirmed that PS and BS fuse together in *Evc2* mutant braincase floors at P8 (Fig. 4*D*, *H*). We then examined the length of the braincase floor at different stages. From E14.5 to E17.5, there were no length differences observed between control and *Evc2* mutant braincase floors (Fig. 4*I*). Starting from E18.5, *Evc2* mutant braincase floors showed shortened length (Fig. 4*I*). More detailed analysis on the length of each region of the braincase floors demonstrated that shortened braincase floor length in *Evc2* mutant was due to shortened anterior region of the braincase floors (Fig. 4*J*), whereas the length of the posterior region of the braincase floors remains unchanged in *Evc2* mutant (Fig. 2*N*).

In summary, histological and morphological analyses of braincase floors at different embryonic stages demonstrate that *Evc2* loss of function more severely impacts the ISS of the braincase floor compared with the SOS of the braincase floor in the following two aspects: (i) chondrocytes in the ISS all become hypertrophic at as early as E18.5 in *Evc2* mutants, whereas a majority of the chondrocytes in the SOS remains undifferentiated; (ii) the overall and anterior part of the braincase floor (PS + ISS + BS) are more severely shortened in *Evc2* mutants after E18.5, whereas the length of the posterior part of the braincase floor (SOS + BO) is less affected after E18.5. Because hypertrophic chondrocytes lose proliferative capacity, premature hypertrophy of the ISS in the *Evc2* mutant braincase floor likely leads to braincase floor shortening due to loss of elongation capacity at the anterior end.

**Fig. 5:**
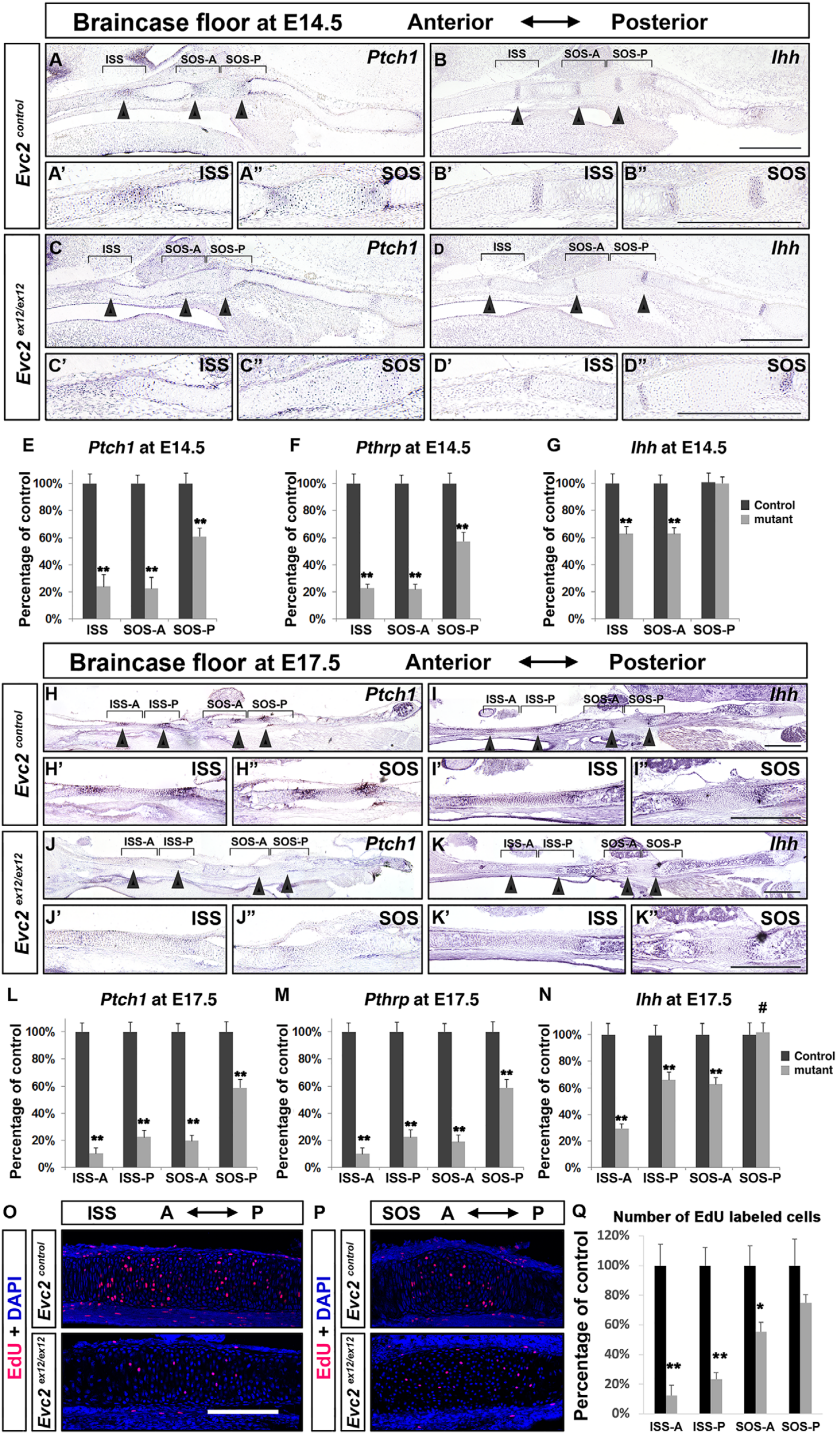
Attenuated hedgehog signaling and proliferation in the *Evc2* mutant braincase floor. Expression of *Ptch1* and *Ihh* were examined in control and *Evc2* mutant braincase floors from E14.5 (*A–D*) and E17.5 (*H–K*). The enlarged images for ISS (*A'–D'*, *H'–K'*) and SOS (*A"–D"*, *H"–K"*) are shown. *Ptch1*, *PthrP*, and *Ihh* expression levels in controls and *Evc2* mutant braincase floors were quantified through qRT‐PCR at E14.5 (*E*–*G*) and E17.5 (*L*–*N*) (*n* = 4, **p < 0.01, #*p* > 0.2). Brackets = regions assayed in qRT‐PCR. Cell proliferation was assessed in E17.5 braincase floor through examination of EdU‐labeled cells after 3 hours of chasing time. EdU‐labeled cells in controls and mutants in ISS (*O*) and SOS (*P*) are shown. Numbers of EdU‐labeled cells were quantified in Q. (*n* = 3, **p < 0.01; *p < 0.05, error bars denote standard deviations.) Scale bar = 200 μm.

Abrogated Hedgehog‐PTHrP signaling leads to accelerated chondrocyte hypertrophy and depleted resting and proliferating chondrocytes.^(^ ^25^, ^26^, ^27^, ^28^ ^)^ Because the abrogated Hedgehog‐PTHrP signaling is the only known reason leading to premature fusion of the growth plate, the premature fusion of the ISS detected in *Evc2* mutant is likely due to decreased Hedgehog signaling. Given that the premature fusion is only detected in the ISS of *Evc2* mutant braincase floors, it is possible to speculate that Hedgehog signaling at the ISS in *Evc2* mutants is much lower than that at the SOS in *Evc2* mutants. Indeed, we observed a lesser decrease in *Ptch1* at the posterior half of the SOS (SOS‐P) but a greater decrease in *Ptch1* at the anterior half of the SOS (SOS‐A) and ISS at E14.5 and at E17.5 *Evc2* mutant braincase floors (Fig. 5*A*, *C*, *E*, *H*, *J*, *L*). Consistently, quantification of *Pthrp* expression levels confirmed greater decrease (relative to the posterior) at the anterior of the *Evc2* mutant braincase floors at E14.5 (Fig. 5*F*) and E17.5 (Fig. 5*M*). PTHRP has a known function to promote chondrocyte proliferation and inhibit chondrocyte differentiation in endochondral ossification. Consistent with more decreased *Pthrp* expression in the anterior of the *Evc2* mutant braincase floor than SOS‐P, we observed highly decreased number of cells with incorporated EdU in the anterior region (Fig. 5*O–Q*). Consistently, we observed highly decreased number of cells with phosphor‐histone 3 (Fig. S3*A*, *C*, *D*) in the anterior region, suggesting a more decreased proliferation in the anterior of the braincase floor.

In *Evc2* mutants at E14.5, we detected no change in *Ihh* expression at the SOS‐P and moderate decreases in *Ihh* at the SOS‐A and the ISS‐P (Fig. 5*B*, *D*). Similar trends were detected at E17.5 (Fig. 5*I*, *K*). As predicted, chondrocytes isolated from *Evc2* mutant braincase floor showed compromised responses to exogenously added smoothened agonist (SAG) in culture judged by *Gli1* expression; however, levels of fold‐induction of Hedgehog signaling activity did not differ between chondrocytes isolated from mutant ISS and mutant SOS (Fig. S3*E*). In appendicular bones, elevated FGF signaling due to *Evc2* loss of function within perichondrium critically contributes to dwarfism.^(^ ^29^ ^)^ However, in the *Evc2* mutant braincase floor, we detected no differences in *Fgf18* expression in the perichondrium compared with controls (Fig. S3*F*). Our data support the idea that premature fusion of the ISS but not the SOS at E18.5 in the *Evc2* mutant braincase floor is due to severely decreased Hedgehog signaling at the ISS yet moderately decreased Hedgehog signaling at the SOS. Taken together, these analyses demonstrated that the progressive reduction of Hedgehog signaling activity along the anterior–posterior axis of the *Evc2* mutant braincase floor is due to differentially decreased *Ihh* expression levels but not due to the position‐based differences in intracellular Hedgehog signaling activating ability.

**Fig. 6:**
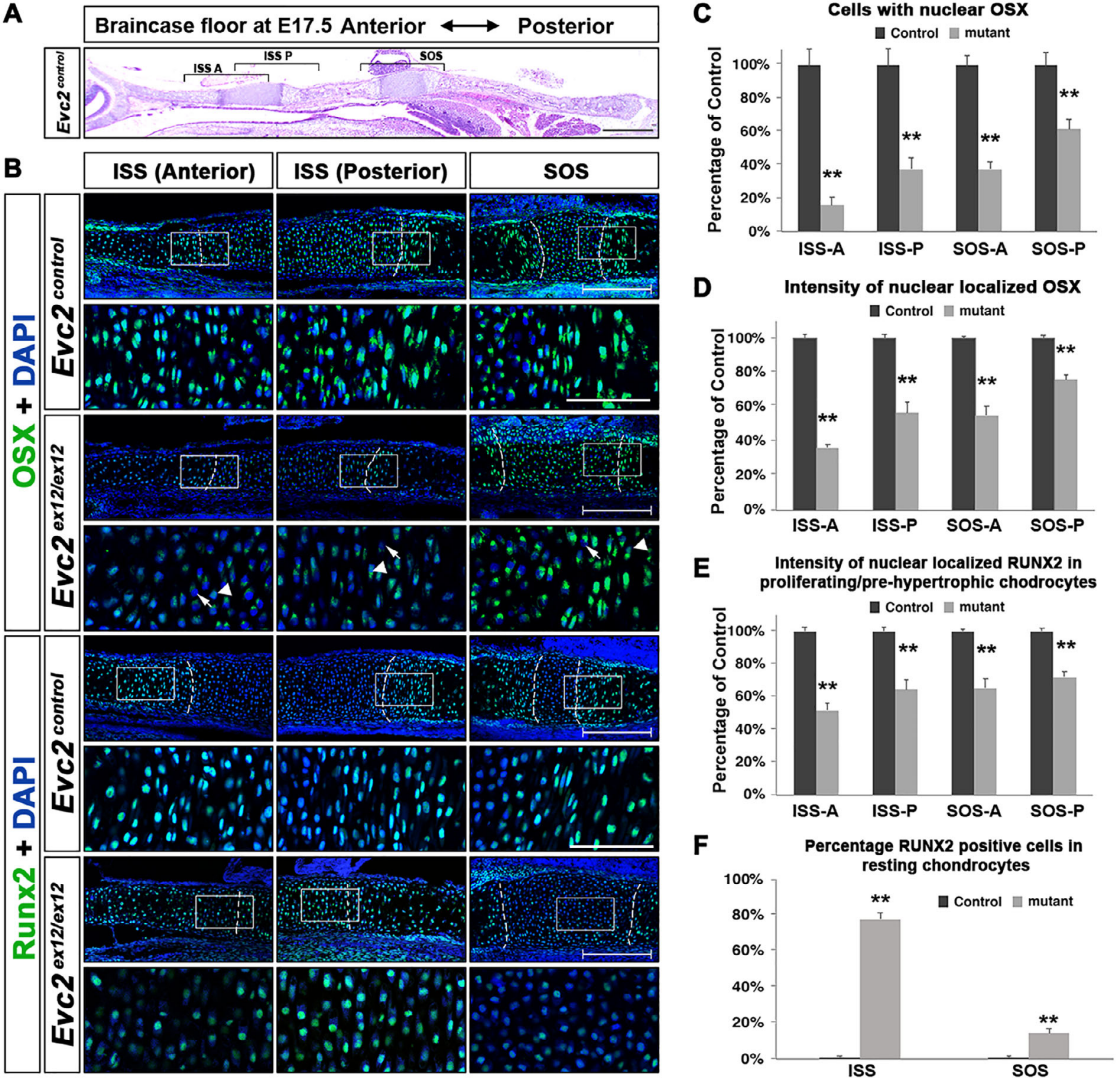
Prehypertrophic differentiation is affected more in the anterior end than the posterior end of the braincase floor. (*A*) A diagram of the braincase floor in the immunohistochemical studies. (*B*) Immunodetection of OSX and RUNX2 in the three areas shown in *A*. The white boxed region was enlarged and shown. Arrows indicate *Evc2* mutant chondrocytes with no nuclear localized OSX; arrowheads indicate *Evc2* mutant chondrocytes with decreased nuclear localized OSX. Scale bar = 200 μm. Dashed lines indicate the boundary between resting and proliferating chondrocytes. (*C*) Number of cells with nuclear localized OSX are quantified and shown as percentage of controls, *n* = 3, **p < 0.01. (*D*) The intensity of nuclear localized OSX was quantified and shown as a percentage of controls, *n* = 4, **p < 0.01, error bars denote standard deviations. (*E*) The intensity of immunosignals for nuclear RUNX2 was quantified and shown as a percentage of control, *n* = 4, **p < 0.01, error bars denote standard deviations. (*F*) The percentages of resting chondrocytes with nuclear localized RUNX2 were quantified and shown, *n* = 3, **p < 0.01. Scale bar = 200 μm, bar in enlarged picture = 20 μm.

*Ihh* is specifically expressed in prehypertrophic chondrocytes but not in hypertrophic chondrocytes within the growth plates. Decreased *Ihh* expression at specific regions of the braincase floor is possibly due to abnormally differentiated prehypertrophic chondrocytes. Osterix (OSX) is a transcription factor with functions during chondrocyte differentiation and maturation.^(^ ^30^, ^31^, ^32^ ^)^ We used OSX to assess the competency of prehypertrophic chondrocyte. In E17.5 braincase floor, we observed cytoplasmic localization in resting (cells with round nucleus in the center of the synchondrosis) and proliferating (cells with oval nucleus in the synchondrosis) chondrocytes and nuclear localization in prehypertrophic and hypertrophic chondrocytes (Fig. 6*A–C*; Fig. S4*A*). Compared with the corresponding regions in controls, *Evc2* mutants showed a decreased number of prehypertrophic chondrocyte cells with nuclear localization of OSX, with a greater decrease at the anterior end of the braincase floor and a lesser decrease at the posterior end of the braincase floor. Similar trends were observed for the intensity of immunosignals of nuclear localized OSX (Fig. 6*B*, *D*). Overall, these data support the idea that compromised differentiation from proliferative chondrocytes to prehypertrophic chondrocytes leads to decreased *Ihh* expression at the SOS‐A and ISS‐P and further decreases at the ISS‐A.

RUNX2 is critical for endochondral ossification.^(^ ^33^, ^34^, ^35^, ^36^ ^)^ At E17.5, RUNX2 located at the nucleus of the proliferating, prehypertrophic, and hypertrophic chondrocytes in both ISS and SOS (Fig. 6*B*). In *Evc2* mutants, we observed decreased intensity of RUNX2 immunosignals at the posterior end and further decreased intensity at the anterior end of the braincase floor (Fig. 6*B*, *E*; Fig. S4*B*). Additionally, in the *Evc2* mutant ISS and SOS, we observed numerous cells in the resting zones with nuclear localized RUNX2 (Fig. 6*B*, *F*), suggesting that the cells in the resting zone were prematurely differentiated to the prehypertrophic/hypertrophic chondrocytes. We did not observe any immunosignals using control IgG (Fig. S4*C*). These observations coincided with the hypertrophy of nearly all cells in the ISS at E18.5 in the *Evc2* mutant braincase floor (Fig. 3*H*) and are consistent with previous studies showing that forced expression of RUNX2 leads to premature hypertrophy of chondrocytes.^(^ ^37^, ^38^, ^39^ ^)^ Overall, these data support the idea that accelerated maturation of resting and proliferative chondrocytes leads to premature hypertrophy of the ISS in the *Evc2* mutant braincase floor.

**Fig. 7:**
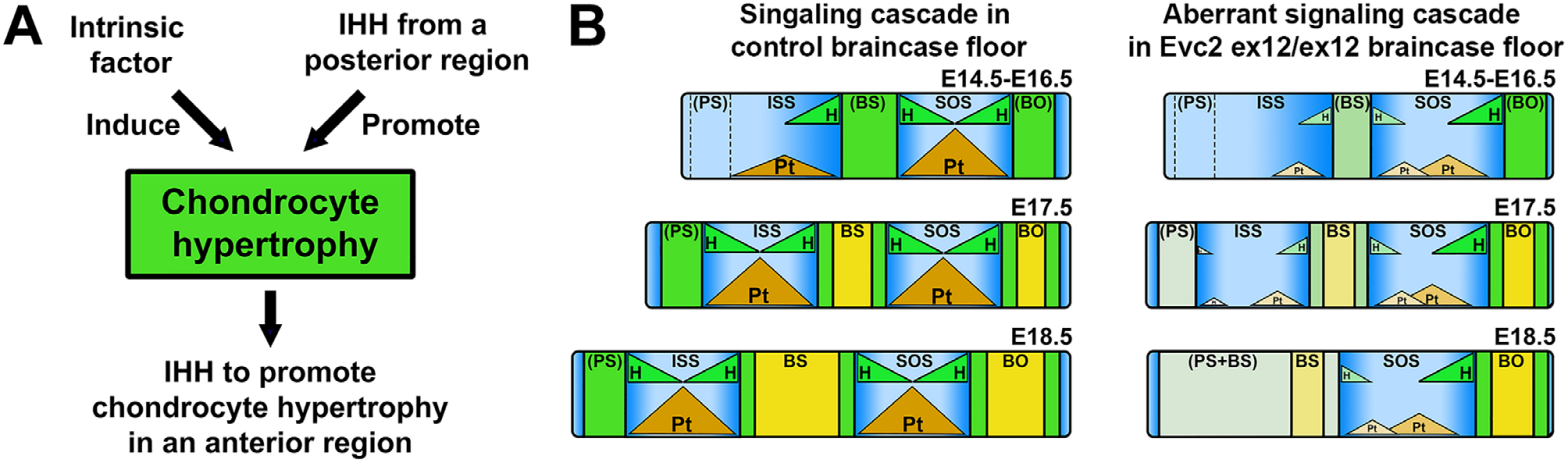
Diagram (*A*) indicated a mechanism governing chondrocyte hypertrophy during braincase floor development. Chondrocyte hypertrophy is induced by an unknown intrinsic factor and promoted by IHH in the prehypertrophic zone from the posterior part. Both intrinsic factor and IHH regulate the chondrocyte hypertrophy and formation of prehypertrophic zone. Formation of the prehypertrophic zone ensures the secretion of IHH, which promotes the chondrocyte hypertrophy at a more anterior region. Diagrams (*B*) indicated the abnormal signaling leading to fusion of the ISS in *Evc2* mutant braincase floor. Three hypertrophic zones in the braincase floor are formed in a sequential order. The formerly formed hypertrophic zone impacts the later formed hypertrophic zone. In the *Evc2* mutant braincase floor, compromised hedgehog signaling at the posterior end progressively reduced anteriorly due to insufficient differentiation to prehypertrophic chondrocytes. Together with reduced cellular response to hedgehog ligands, expression of *Pthrp* is largely diminished at the ISS, which leads to the premature fusion of the ISS. Pt = *Pthrp*; H = *Ihh*.

Chondrocyte hypertrophy can occur in the absence of Hedgehog signaling; however, studies in appendicular bones suggest that Hedgehog signaling promotes chondrocyte hypertrophic differentiation.^(^ ^36^, ^40^, ^41^, ^42^ ^)^ Given that chondrocyte hypertrophy occurs through a sequential manner from the posterior part to the anterior part, it is therefore possible that the chondrocyte hypertrophy in the braincase floor is induced by an unknown intrinsic factor and promoted by IHH secreted from the posterior hypertrophic zone (Fig. 7*A*). Particularly in the *Evc2* mutant braincase floors, compromised Hedgehog signaling in the SOS‐P leads to insufficient hypertrophic differentiation in the hypophyseal cartilage (BS), which results in abnormalities in prehypertrophic chondrocytes at the SOS‐A and ISS‐P. Decreased *Ihh* expression in the ISS‐P and reduced cellular response to Hedgehog ligand due to *Evc2* loss of function together leads to highly decreased Hedgehog signaling in the ISS‐P; this reduction has a greater impact on hypertrophic differentiation in the trabecular cartilage and prehypertrophic chondrocytes at the ISS‐A. The above described signaling cascade in the *Evc2* mutant braincase floor results in drastically decreased Hedgehog signaling in the ISS along with a drastic reduction of *Pthrp* expression, prompting chondrocyte hypertrophic differentiation and subsequent premature ISS fusion (Fig. 7*B*).

**Fig. 8:**
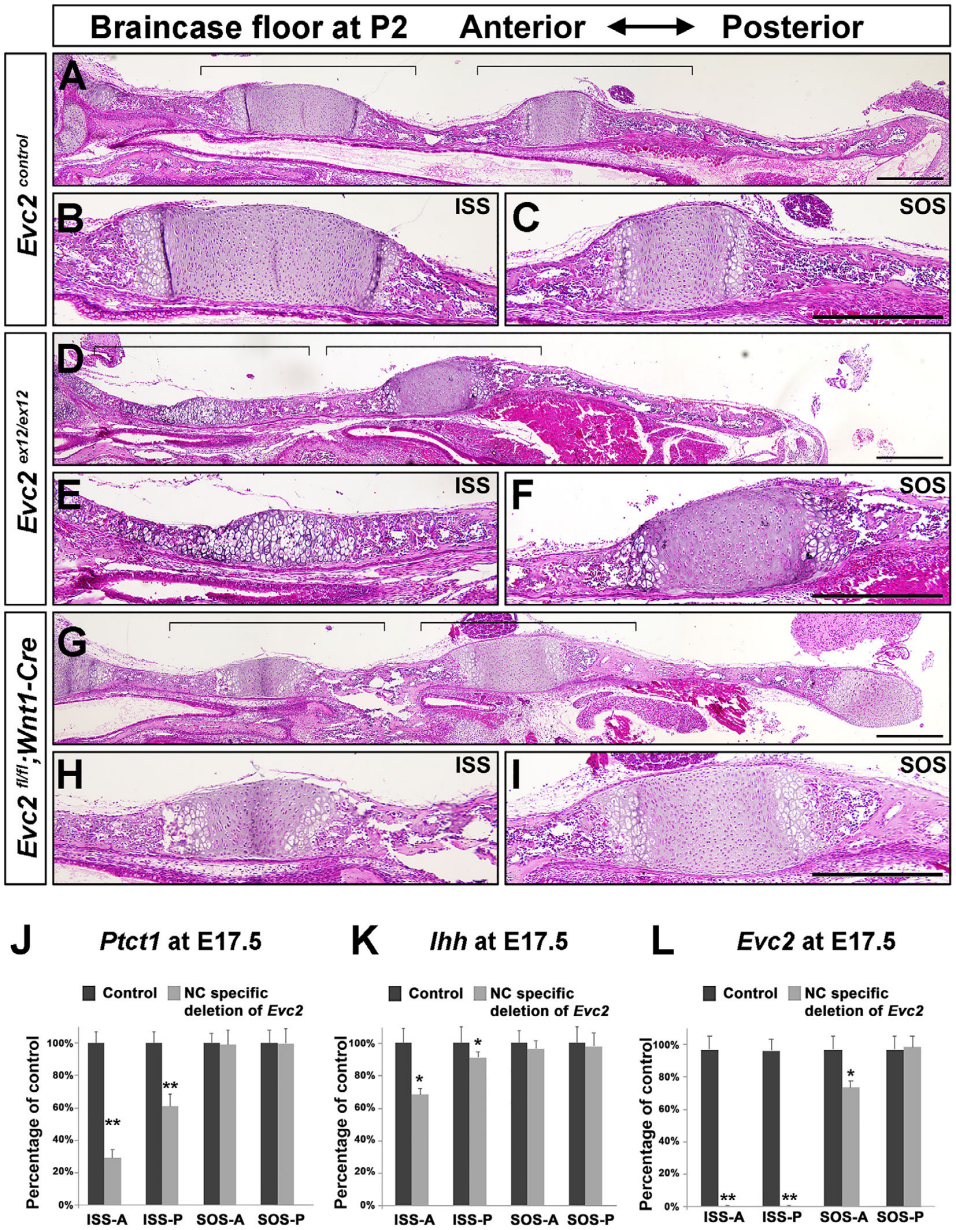
Specific deletion of *Evc2* in neural crest–derived tissues leads to patent ISS at P2. Control (*A*), *Evc2* mutant (*D*), and *Evc2 Wnt1* mutant (*G*) braincase floor at P2 were sagittal sectioned and stained with H&E. The enlarged ISS and SOS for control (*B*, *C*), *Evc2* mutant (*E*, *F*), and *Evc2‐Wnt1‐Cre* conditional mutants (*H*, *I*) are shown. Brackets indicate regions enlarged. Scale bar = 200 μm. *Ptch1* (*J*), *Ihh* (*K*), and *Evc2* (*L*) expression levels in controls and *Evc2‐Wnt1‐Cre* conditional mutant braincase floors were assayed through qRT‐PCR at E17.5. (*n* = 4, **p < 0.01, error bars denote standard deviations.)

From the above interpretation, the drastic decreased Hedgehog signaling in the ISS of the *Evc2* mutant braincase floor resulted from a locally compromised response to Hedgehog ligands due to *Evc2* loss of function, and decreased *Ihh* expression in the ISS due to a spatio impact from the *Evc2* loss of function within the parachordal cartilage. The compromised response to Hedgehog ligands due to *Evc2* loss of function has been demonstrated previously from our group and others.^(^ ^20^, ^29^, ^43^ ^)^ To validate the spatio impact of *Evc2* loss of function in the parachordal cartilage, we took advantage of *Wnt1‐Cre* to restrict *Evc2* deletion to the anterior of the SOS‐A (Fig. S1*I*).^(^ ^10^ ^)^ As expected, the ISS remains present in *Evc2‐Wnt1‐Cre* conditional mutants at P2 (Fig. 8*G–H*). In contrast, the whole ISS in *Evc2* global mutants became hypertrophic (Fig. 8*D–E*). Gene expression analyses demonstrated that compared with *Evc2* mutants, *Evc2‐Wnt1‐Cre* conditional mutants showed moderately decreased Hedgehog signaling levels and slightly decreased *Ihh* expression at the ISS (Fig. 8*J–K*). Examination of *Evc2* expression in *Evc2‐Wnt1‐Cre* conditional mutants braincase floors confirms the deletion of *Evc2* in the ISS (Fig. 8*L*). These data demonstrate that *Evc2* function in the parachordal cartilage of the braincase floor is critical for the trabecular cartilage development of the braincase floor and support the idea that hypertrophy of the parachordal cartilage of the braincase floor subsequently impacts the hypertrophy of the hypophyseal cartilage and trabecular cartilage.

## Discussion

Overall, our analysis of braincase floor development in controls and *Evc2* mutants demonstrated that: (i) hypertrophy of chondrocytes in the braincase floor cartilage occurs in a sequential, posterior‐to‐anterior manner (ie, parachordal, then hypophyseal, then trabecular cartilage); (ii) chondrocyte hypertrophy at the parachordal cartilage of the braincase floor impacts hypertrophy of the hypophyseal and trabecular cartilage of the braincase floor; (iii) loss of proliferative capacity in the *Evc2* mutant ISS is determined at E18.5; (iv) loss of proliferative capacity in the *Evc2* mutant ISS is due to progressive spatiotemporal reduction of Hedgehog signaling during the posterior to the anterior hypertrophy of braincase floor chondrocytes.

The braincase floor is a critical midline structure, which directly impacts protrusion of the midfacial region. However, braincase floor elongation is not homogeneous. The posterior braincase floor grows slower, and the anterior braincase floor grows faster.^(^ ^44^, ^45^ ^)^ Fusion of the ISS occurs between 2 and 3 years of age in humans.^(^ ^46^ ^)^ Since the ISS significantly contributes to embryonic and early postnatal braincase floor elongation, premature fusion of the ISS would result in congenital midfacial hypoplasia.

The findings in presented studies are consistent with previous studies about the Hedgehog signaling during endochondral ossifications. Hedgehog signaling plays multiple roles in endochondral ossifications, including promoting proliferation of resting chondrocyte through PTHrP,^(^ ^47^ ^)^ directly promoting differentiation of resting chondrocyte to proliferating chondrocyte,^(^ ^41^ ^)^ and promoting differentiation from proliferating chondrocyte to prehypertrophic chondrocyte and to hypertrophic chondrocyte.^(^ ^42^ ^)^ The impacts of decreased Hedgehog signaling in endochondral ossification can be categorized into two: (i) When Hedgehog signaling is largely attenuated, decreased *Pthrp* (direct Hedgehog signaling target) expression leads to insufficient proliferation in resting chondrocyte, which leads to depletion of resting chondrocyte and premature fusion of growth plate.^(^ ^47^ ^)^ Studies in appendicular bones demonstrated that ablating primary cilium leads to attenuation of Hedgehog signaling to 15% of controls, which subsequently leads to premature fusion of the growth plate.^(^ ^48^, ^49^ ^)^ (ii) When Hedgehog signaling is moderately decreased due to loss of *Evc2* functions, chondrocyte proliferation and maturation is delayed, while growth plates remain.^(^ ^29^ ^)^ Studies using *Evc2* mutant mice demonstrated that attenuating Hedgehog signaling to 50% of controls leads to delayed chondrocyte differentiation. In this case, bone maturation takes longer time but eventually fully form the primordia for further ossification to occur.^(^ ^29^ ^)^ Consistent with what happens in appendicular bones, we found that the moderately decreased Hedgehog signaling (50% of controls) in the SOS of *Evc2* mutant braincase floor leads to limited decreased chondrocyte proliferation and temporally decreased length in BO. The shortened length in BO later catches up the length of BO in controls at postnatal stages day 8. In contrast, dramatically decreased Hedgehog signaling (10% of controls) in the ISS of *Evc2* mutant braincase floors leads to dramatically decreased chondrocyte proliferation and depletion of chondrocyte capable of proliferation. The dramatic decreased Hedgehog signaling in the ISS of *Evc2* mutant braincase floor favors the cascade mechanism during braincase floor development as summarized in Figs. 1*B* and 7*B*. Consistently, in *Evc2‐Wnt1‐Cre* conditional mutants, we observed no premature fusion of ISS at up to P2 and moderately decreased Hedgehog signaling and moderately decreased *Ihh* expression. These observations well support the cascade mechanism we proposed.

Our studies support a mechanism that during braincase floor development, the hypertrophy of each zone is induced by two factors, an unknown intrinsic factor and IHH secreted from immediate posterior region (Fig. 7*A*). The unique two factor‐involved chondrocyte hypertrophy supports a cascade mechanism to explain how the posterior hypertrophy influences the anterior hypertrophy during braincase floor development. The initial chondrocyte hypertrophy occurs at the parachordal cartilage of the braincase floor, next to the notochord remnants to develop SOS‐P.^(^ ^14^ ^)^ Hypertrophy of the hypophyseal cartilage is induced by an intrinsic factor and promoted by *Ihh* secreted by SOS‐P. The hypertrophy of the hypophyseal cartilage leads to formations of prehypertrophic zones at the SOS‐A and ISS‐P. Similarly, the hypertrophy of the trabecular cartilage is induced by an intrinsic factor and promoted by *Ihh* secreted by prehypertrophic zones (Fig. 7*B*). In the *Evc2* mutant braincase floor, compromised response to *Ihh* at the SOS‐P leads to insufficient hypertrophic differentiation and abnormally formed prehypertrophic chondrocytes. Decreased *Ihh* in abnormally formed prehypertrophic chondrocytes then leads to further decreased Hedgehog signaling at the ISS, which leads to premature hypertrophy of chondrocytes at the ISS due to highly reduced PTHrP and subsequent loss of elongation capacity, as shown in Fig. 7*B*. The sequential hypertrophy of the parachordal, then the hypophyseal, then the trabecular cartilage in the braincase floor cascade model provides critical insights into the pathological mechanism leading to shortened anterior braincase floor in EvC and other syndromes such as Apert syndrome, Pfeiffer syndrome, Crouzon syndrome, Down syndrome, and William syndrome.

## Disclosures

The authors declared no potential conflicts of interest with respect to the research, authorship, and/or publication of this article.

### Peer Review

The peer review history for this article is available at https://publons.com/publon/10.1002/jbm4.10589.

## Supporting information


**Fig. S1.** Wild‐type (*A*) and *Evc2* heterozygous mutant (*D*) braincase floors were stained for beta‐galactosidase activity. The ISS and SOS of wild‐type (*B*, *C*) and heterozygous mutant (*E*, *F*) are enlarged. Cre‐dependent recombination in the braincase floor of *Wnt1‐Cre* lines are indicated (*G*). The ISS and SOS (*H*, *I*) are enlarged. Scale bar = 200 um.

**Fig. S2.** (*A*) Control and *Evc2* mutant braincase floors at E13.5 were sagittally sectioned and stained with H&E. Arrows indicate the gap between the trabecular cartilage and the other three cartilages (the hypophyseal, acrochordal and parachordal, cartilages) in the braincase floor. (*B*) Quantification of *Evc2* mRNA in the indicated tissues at the indicated stages. T = the trabecular cartilage; H‐P = from the hypophyseal to the parachordal cartilage; ISS = the intersphenoid synchondrosis; SOS = the spheno‐occipital synchondrosis.

**Fig. S3.** Cell proliferation was assessed through examination of chondrocytes with phospho‐histone 3 (P‐Histone 3) in controls and mutants in ISS (*A*) and SOS (*B*). White arrows indicate cells with P‐Histone 3. Boxed regions are enlarged and shown. Numbers of cells with P‐Histone 3were quantified in *C* as percentage of control (*n* = 4, **p < 0.01; #*p* > 0.2, error bars denote standard deviations). Scale bar = 200 um. Actual numbers of cells with P‐Histone 3 are shown in *D*. (*n* = 4, **p < 0.01; error bars denote standard deviations.) (*E*) Primary chondrocytes were isolated from ISS and SOS in control (C ISS and C SOS) and *Evc2* mutant (m ISS and m SOS) braincase floors at E17.5. Smoothened agonist (SAG) was used to treat each type of primary chondrocyte cells. The *Gli1* mRNA levels from each type of treated cells were used to readout the induced Hedgehog signaling levels. (*n* = 3, **p < 0.01, #*p* > 0.4, error bars denote standard deviations.) (*F*) *Fgf18* expression in the E17.5 braincase floor was examined through in situ hybridization. Scale bar = 200 um.

**Fig. S4.** (*A*) Number of cells with nuclear localized OSX are quantified and shown, *n* = 3, **p < 0.01, error bars denote standard deviations. (*B*) The percentages of resting chondrocytes with nuclear localized RUNX2 were quantified and shown, *n* = 3, **, *p* < 0.01. Scale bar = 200 um, bar in enlarged picture = 20 um, error bars denote standard deviations. (*C*) Immunodetection using non‐specific IgG controls in control braincase floor was shown.

## Data Availability

Data available on request from the authors.
