# Supplementary figures and images for: The Posterior Part Influences the Anterior Part of the Mouse Cranial Base Development
Source: JBMR Plus. 2021 Dec 24;6(2):e10589. doi: 10.1002/jbm4.10589 (PMC8861986; doi:10.1002/jbm4.10589)

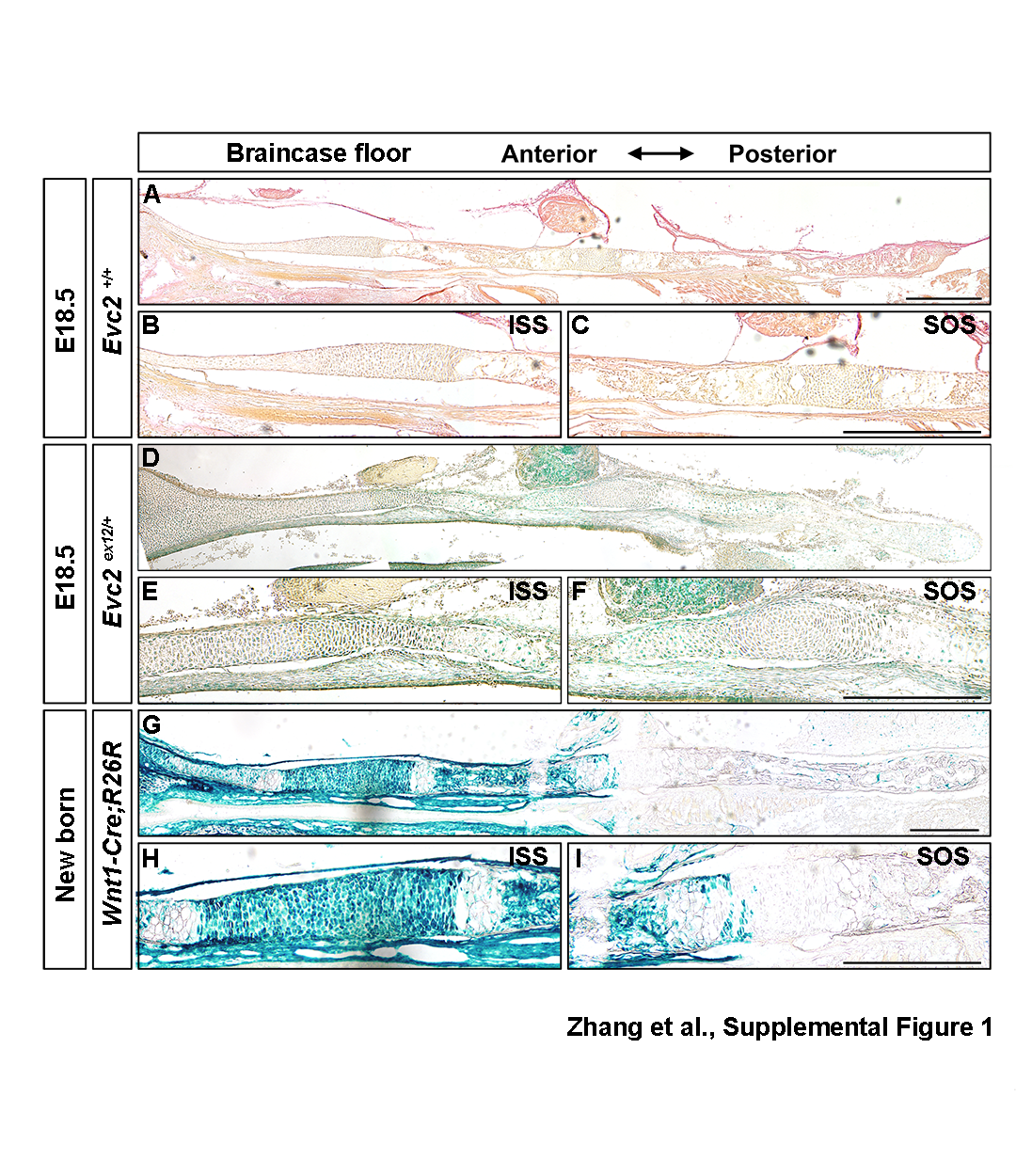

Supplement: Supplementary file 1 — Fig. S1. Wild‐type (A) and Evc2 heterozygous mutant (D) braincase floors were stained for beta‐galactosidase activity. The ISS and SOS of wild‐type (B, C) and heterozygous mutant (E, F) are enlarged. Cre‐dependent recombination in the braincase floor of Wnt1‐Cre lines are indicated (G). The ISS and SOS (H, I) are enlarged. Scale bar = 200 um. [file JBM4-6-e10589-s003.tif]

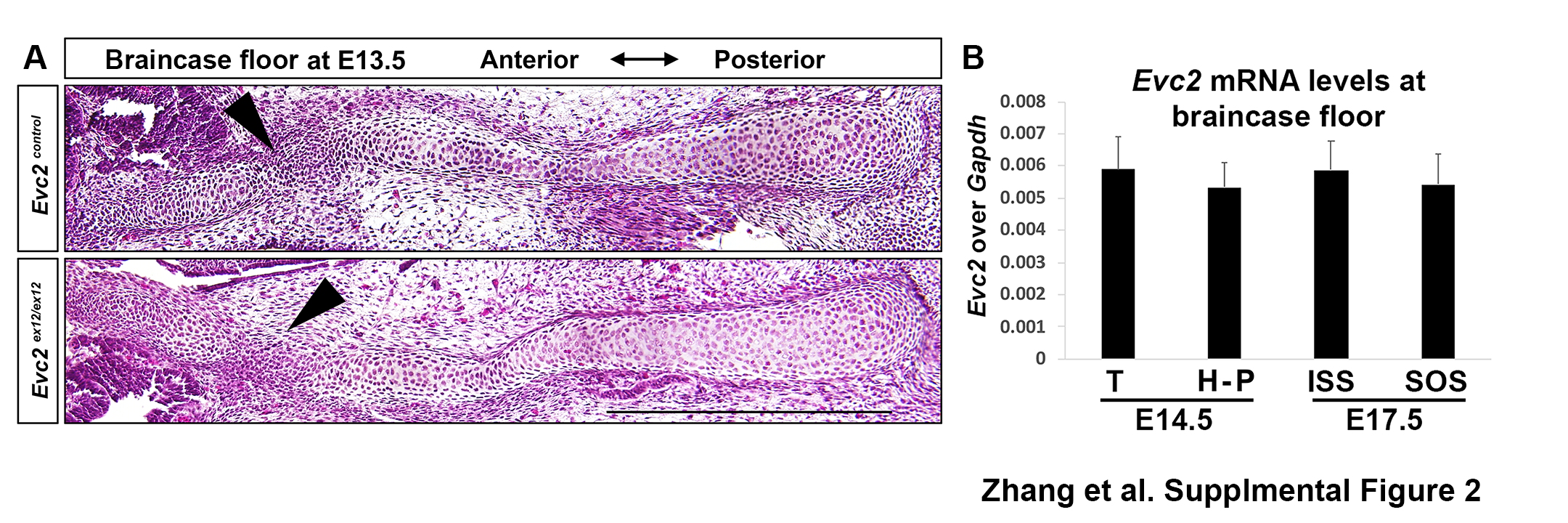

Supplement: Supplementary file 2 — Fig. S2. (A) Control and Evc2 mutant braincase floors at E13.5 were sagittally sectioned and stained with H&E. Arrows indicate the gap between the trabecular cartilage and the other three cartilages (the hypophyseal, acrochordal and parachordal, cartilages) in the braincase floor. (B) Quantification of Evc2 mRNA in the indicated tissues at the indicated stages. T = the trabecular cartilage; H‐P = from the hypophyseal to the parachordal cartilage; ISS = the intersphenoid synchondrosis; SOS = the spheno‐occipital synchondrosis. [file JBM4-6-e10589-s001.tif]

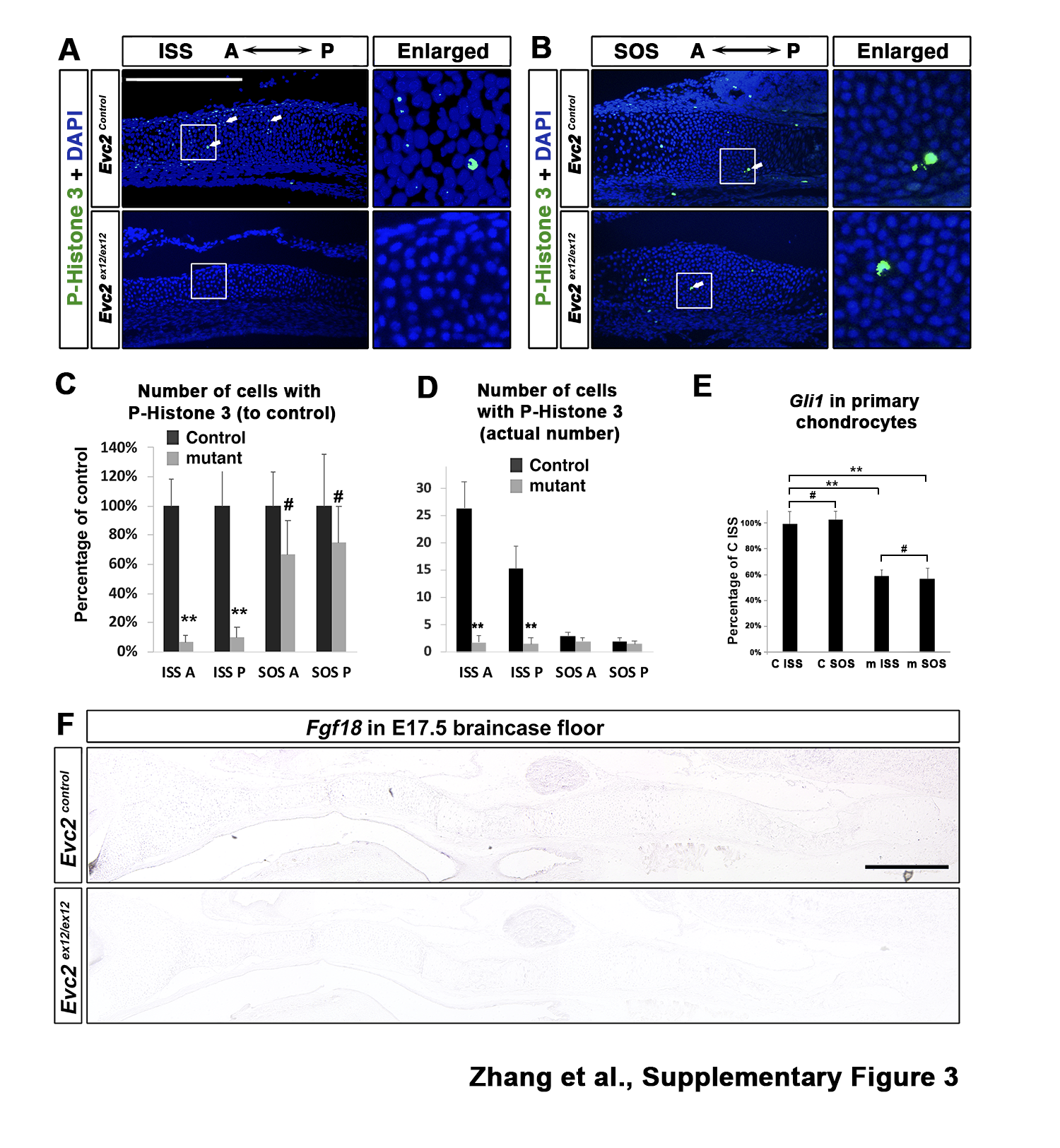

Supplement: Supplementary file 3 — Fig. S3. Cell proliferation was assessed through examination of chondrocytes with phospho‐histone 3 (P‐Histone 3) in controls and mutants in ISS (A) and SOS (B). White arrows indicate cells with P‐Histone 3. Boxed regions are enlarged and shown. Numbers of cells with P‐Histone 3were quantified in C as percentage of control (n = 4, **p < 0.01; #p > 0.2, error bars denote standard deviations). Scale bar = 200 um. Actual numbers of cells with P‐Histone 3 are shown in D. (n = 4, **p < 0.01; error bars denote standard deviations.) (E) Primary chondrocytes were isolated from ISS and SOS in control (C ISS and C SOS) and Evc2 mutant (m ISS and m SOS) braincase floors at E17.5. Smoothened agonist (SAG) was used to treat each type of primary chondrocyte cells. The Gli1 mRNA levels from each type of treated cells were used to readout the induced Hedgehog signaling levels. (n = 3, **p < 0.01, #p > 0.4, error bars denote standard deviations.) (F) Fgf18 expression in the E17.5 braincase floor was examined through in situ hybridization. Scale bar = 200 um. [file JBM4-6-e10589-s002.tif]

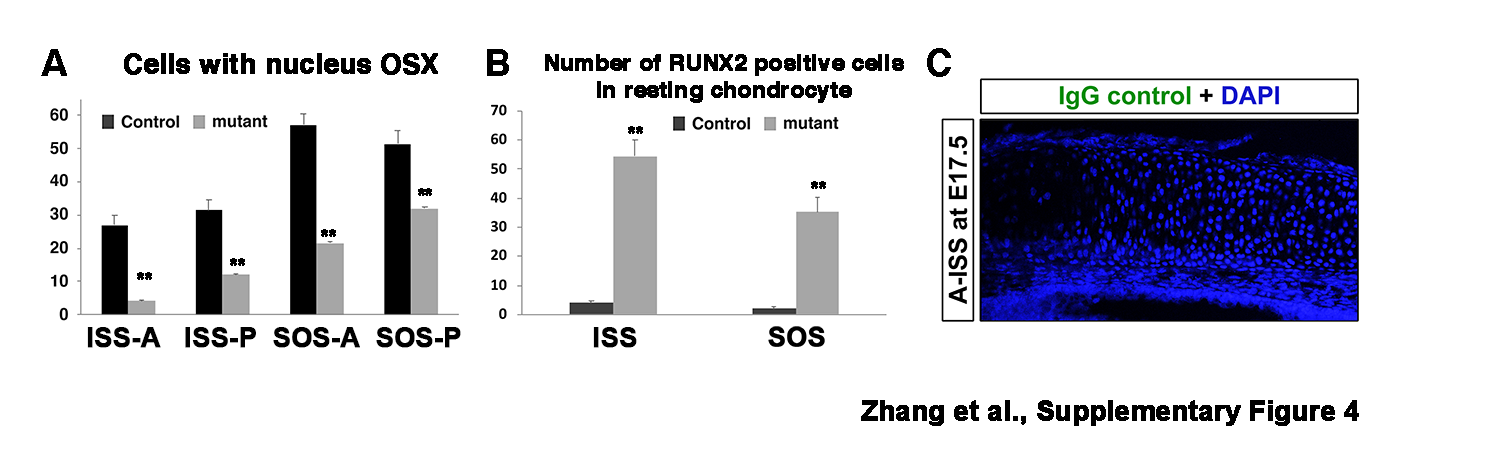

Supplement: Supplementary file 4 — Fig. S4. (A) Number of cells with nuclear localized OSX are quantified and shown, n = 3, **p < 0.01, error bars denote standard deviations. (B) The percentages of resting chondrocytes with nuclear localized RUNX2 were quantified and shown, n = 3, **, p < 0.01. Scale bar = 200 um, bar in enlarged picture = 20 um, error bars denote standard deviations. (C) Immunodetection using non‐specific IgG controls in control braincase floor was shown. [file JBM4-6-e10589-s004.tif]
